# From compartments to loops: understanding the unique chromatin organization in neuronal cells

**DOI:** 10.1186/s13072-024-00538-6

**Published:** 2024-05-23

**Authors:** Diana Zagirova, Anna Kononkova, Nikita Vaulin, Ekaterina Khrameeva

**Affiliations:** 1 Center for Molecular and Cellular Biology, Skolkovo Institute of Science and Technology, Bolshoy Boulevard 30, Build.1, Moscow, 121205 Russia; 2 Research and Training Center on Bioinformatics, Institute for Information Transmission Problems (Kharkevich Institute) RAS, Bolshoy Karetny per. 19, Build.1, Moscow, 127051 Russia

**Keywords:** Chromatin, Brain, Neurons, Glia, Hi-C

## Abstract

The three-dimensional organization of the genome plays a central role in the regulation of cellular functions, particularly in the human brain. This review explores the intricacies of chromatin organization, highlighting the distinct structural patterns observed between neuronal and non-neuronal brain cells. We integrate findings from recent studies to elucidate the characteristics of various levels of chromatin organization, from differential compartmentalization and topologically associating domains (TADs) to chromatin loop formation. By defining the unique chromatin landscapes of neuronal and non-neuronal brain cells, these distinct structures contribute to the regulation of gene expression specific to each cell type. In particular, we discuss potential functional implications of unique neuronal chromatin organization characteristics, such as weaker compartmentalization, neuron-specific TAD boundaries enriched with active histone marks, and an increased number of chromatin loops. Additionally, we explore the role of Polycomb group (PcG) proteins in shaping cell-type-specific chromatin patterns. This review further emphasizes the impact of variations in chromatin architecture between neuronal and non-neuronal cells on brain development and the onset of neurological disorders. It highlights the need for further research to elucidate the details of chromatin organization in the human brain in order to unravel the complexities of brain function and the genetic mechanisms underlying neurological disorders. This research will help bridge a significant gap in our comprehension of the interplay between chromatin structure and cell functions.

## Background

**Fig. 1:**
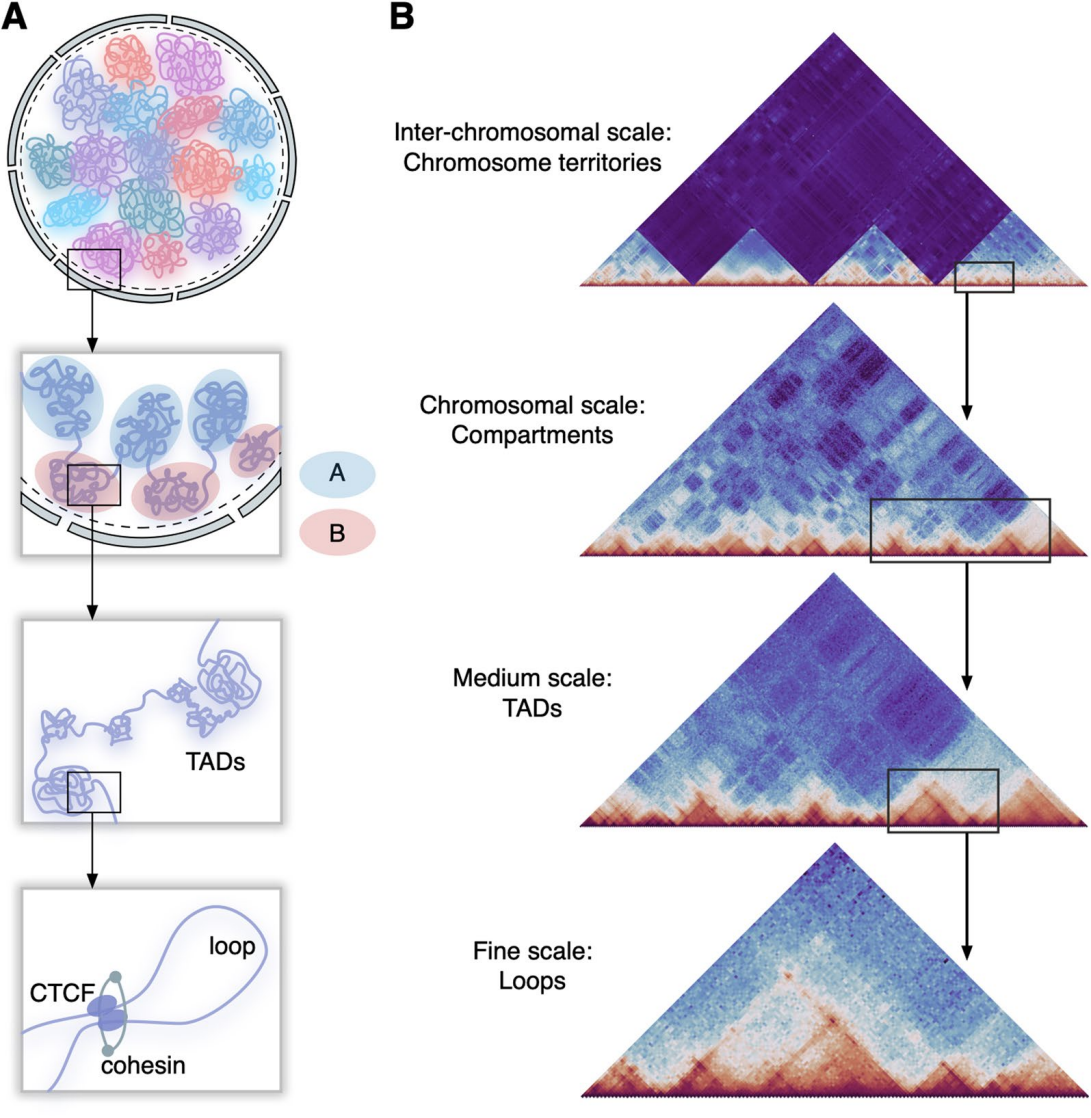
General principles of chromatin organization. **A** Schematic representation of the major levels of chromatin organization in the nucleus: chromosome territories, compartments A and B, topologically associated domains (TADs), and chromatin loops. **B** Levels of chromatin organization in the Hi-C data. The intensity of pixels on Hi-C maps is proportional to the contact frequencies

Mammalian genomes are characterized by a complex 3D architecture with many levels of organization, representing a multilayered system with a specific functionality. With the development of chromosome conformation capture (3C) technology, it was discovered that eukaryotic genomes are organized hierarchically [1]. From the large to small scale, the nucleus contains chromosomal territories, chromatin compartments, topologically associated domains (TADs), chromatin loops and nucleosomes (Fig. 1A). Individual chromosomes are clearly separated in the three-dimensional space of the nucleus, leading to the formation of chromosome territories—nuclear regions predominantly occupied by different interphase chromosomes [2]. Data obtained using high-throughput chromosome conformation capture technology (Hi-C, Fig. 1B) show that chromosomes are further divided into two compartments. Compartment A, which is usually located in the center of the nucleus, consists of active epigenetic marks and actively transcribed genes. Compartment B, located adjacent to the nuclear lamina, consists of repressive epigenetic marks and inactive genes [3]. The formation of compartments A and B is thought to result from a combination of factors, including the distribution of active and repressive chromatin marks [4].

In addition to chromatin compartments, there is another level of the genome organization formed by TADs—genomic regions with frequent interactions within them and high isolation from neighboring domains [5]. TADs are proposed to be the major functional regulatory domains that modulate contacts between enhancers and promoters. An increased frequency of contacts within TADs facilitates physical interactions between enhancer-promoter pairs, while strong insulation at TAD boundaries limits such interactions for enhancer-promoter pairs located in adjacent TADs. TAD boundaries are highly stable between species and are enriched with CCCTC binding factor (CTCF) and cohesin in mammals. CTCF and cohesin form chromatin loops that anchor TAD boundaries, providing a structural basis for TAD formation. Chromatin loops are thought to form through a loop extrusion model based on the interaction of CTCF and cohesin complex [6]. Physical interactions between distal regions of the genome allow linearly spaced elements such as promoters and enhancers to encounter each other. Long-range contacts between enhancers and promoters can be modulated by transcription factors, Mediator complex, RNA polymerase II and non-coding RNAs, which further regulate gene expression [7].

Contacts mediated by Polycomb Group (PcG) proteins represent another special level of three-dimensional genome organization. PcG proteins form two multi-subunit Polycomb repressive complexes: PRC1 and PRC2 [8]. PRC1 and PRC2 are responsible for introducing and recognizing chromatin modifications H2AK119ub1 and H3K27me3, respectively. These complexes operate reciprocally, with the product of one complex serving as a substrate for the other [8, 9]. Experiments examining the spatial structure of chromatin provide evidence for the significant impact of PcG proteins on DNA packaging, as two studies have reported the formation of Polycomb-mediated TADs in mice [10, 11]. However, a defining characteristic of PcG proteins is their ability to mediate long-range DNA interactions, as demonstrated in *Drosophila*, mice, and humans [12–15]. Therefore, PcG proteins act as crucial regulators of chromatin architecture, complementing the traditional chromatin organization units by mediating long-range interactions.

Although the three-dimensional structure of chromatin is globally stable, recent studies indicate that individual genes frequently switch between active and inactive compartments during development. In addition, specific interactions within and outside TADs often change [16]. The observed associations between chromatin compartmentalization, TAD profile, and transcriptional activity indicate possible causal relationships between them. Accordingly, a comparative analysis of Hi-C maps in the fruit fly *Drosophila* before and after transcriptional suppression provides insights into the interplay between chromatin structure and gene expression. This analysis reveals that the three-dimensional organization of chromatin not only regulates gene activity, but is also influenced by genes involved in transcriptional processes [17, 18]. Additionally, a complex architecture of chromatin within the nucleus plays a pivotal role in the regulation of diverse cellular functions and in maintaining the integrity of the genome.

Considering the tight connection of the genome architecture with gene expression, unveiling details of the genome organization could elucidate the mechanisms underlying tissue-specific properties, as well as advance understanding of pathological processes leading to diseases. Therefore, Hi-C studies focused on the brain tissue are currently among the most demanding ones due to the limited understanding of the complex processes involved in brain development, function and degenerative changes. Few studies have explored the differences in genome architecture between neurons and various non-neuronal cells in the human or mouse brain [19–23]. These studies have laid the foundation for chromatin research in the brain by demonstrating that while brain cells generally conform to the fundamental principles of three-dimensional genome organization, they also exhibit unique features of chromatin architecture at several structural levels.

## Main text

### Compartmentalization in neurons and non-neuronal brain cells

**Fig. 2:**
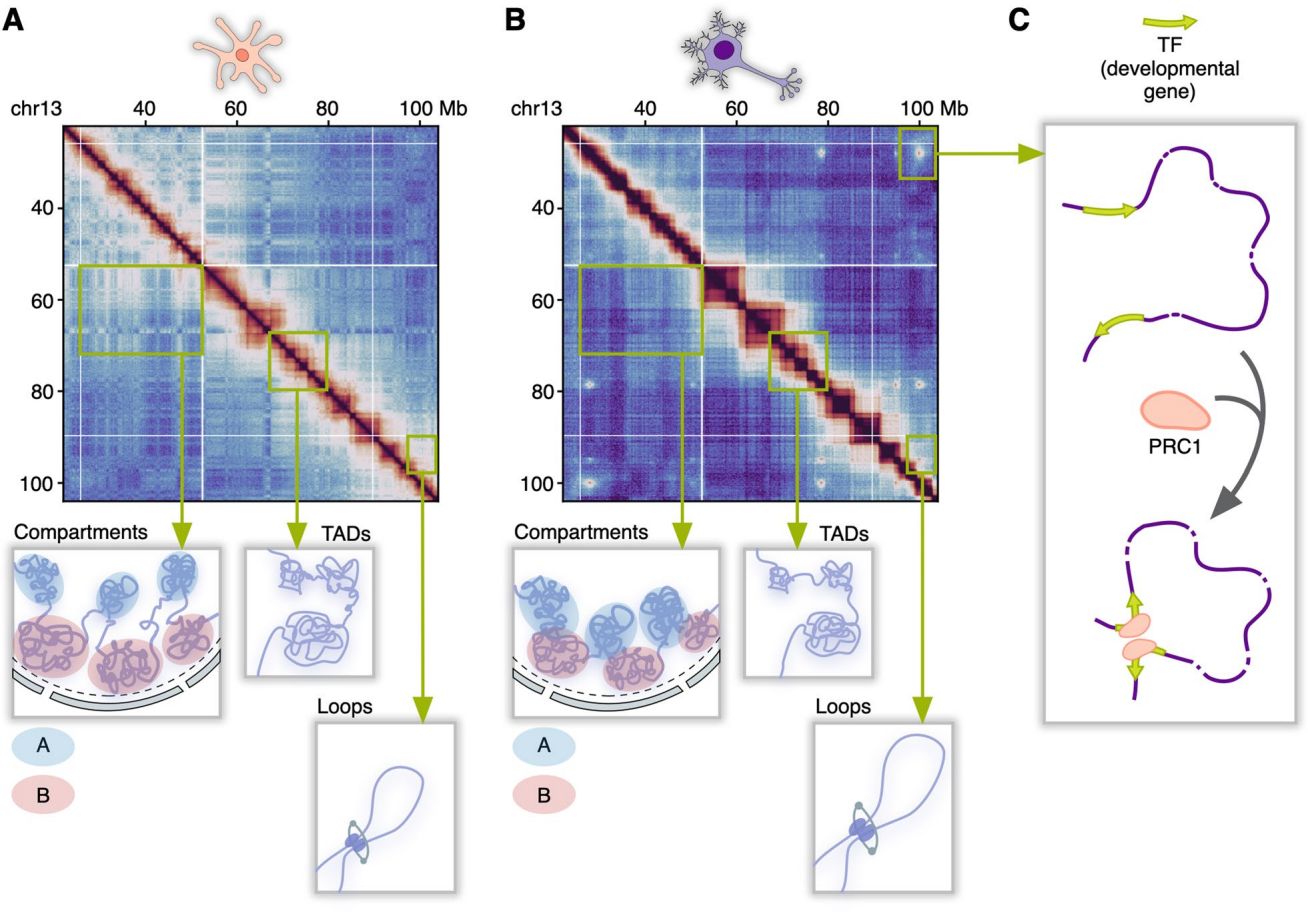
Differences in chromatin organization between neuronal and non-neuronal brain cell types at various chromatin organization levels. **A** The main features of chromatin organization in non-neuronal brain cells: strong compartmentalization, a lower density of TADs, and shorter chromatin loops. **B** Characteristics of chromatin organization in neurons: weaker compartmentalization, an increased density of TADs, and extended chromatin loops. **C** A hallmark of neuronal chromatin organization: neuron-specific long-range chromosomal interactions mediated by Polycomb Group (PcG) proteins

Most studies investigating the chromatin architecture in the brain focus on comparing neurons with other cell types that lack a neuronal marker Neuronal Nuclear Antigen (NeuN). This mixture includes glial cells, such as oligodendrocytes, astrocytes, microglia, ependymal cells, as well as other cell types like endothelial cells. We will collectively refer to these cells as NeuN-negative cells for simplicity. One such study by Rahman et al. has demonstrated that neurons have a distinct chromatin structure at a large-scale level, characterized by a weaker compartmentalization compared to other types of brain cells [19] (Fig. 2A, B). Moreover, within the organization of A/B compartments, neurons display unique features. Specifically, neurons exhibit elevated levels of short-range A–A interactions, while long-range B–B contacts are reduced in neurons compared to NeuN-negative cells.

The difference in compartment strength is not likely explained by the heterogeneity of NeuN-negative cells, as microglia, a specific glial cell type, exhibits the same trend. Furthermore, recent research on the genomic architecture at the single-cell level supports these observations, with neurons showing more pronounced short-range chromosomal interactions [21]. Conversely, a variety of NeuN-negative cell types exhibit an increase in long-range intra-compartment contacts and a decrease in inter-compartment interactions, indicating enhanced compartment strength.

The differences observed in compartmentalization features may be attributed to the increased loop extrusion in neurons. Several studies have indicated that cohesin complexes, which play a pivotal role in loop extrusion, together with CTCF, influence the regulation of A/B compartment interactions at both short- and long-range levels. For instance, depleting a specific type of cohesin complex leads to an increase in long-range interactions among regions located in B compartments, along with a substantial decrease in mid-range interactions among A compartments [24]. Another study on the cohesin-loading factor *Nipbl* KO in mouse liver has shown that cohesin depletion leads to a reduction in the intensity of TADs while accentuating compartmentalization [25]. This pattern is similar to that observed when comparing NeuN-negative cells to neurons. Moreover, this study has revealed that *Nipbl* depletion leads to the down-regulation of genes located in regions with extended intergenic spaces, which are notably more prevalent in neurons. Indeed, studies suggest that certain neuronal genes, particularly those expressed in multiple types of neurons, reside in large, gene-poor, non-coding regions that contain a high number of regulatory elements, which could potentially participate in establishing the gene expression program across diverse neuronal cells [26, 27].

Collectively, these findings suggest that the distinct chromatin architecture in neurons may be partially due to elevated levels of cohesin or its associated proteins. Although comprehensive evidence for this is still lacking, the work of Rahman et al. indicates a slight increase in the expression of the cohesin complex protein RAD21 in neurons [19], which is known to play a crucial role in loop extrusion [28]. Thus, despite the general pattern derived from Hi-C data suggesting that loop extrusion may be more prevalent in neurons compared to NeuN-negative cells, there is only limited evidence indicating an increased presence of cohesin-related proteins in neurons. Further research is required to determine the exact extent of cohesin’s impact on chromatin structure in neurons, including its potential role in reducing compartmentalization.

Despite an overall decrease in strength of the neuronal compartment B, a detailed investigation in the mouse cortex has revealed a distinct neuron-specific inactive subcompartment exhibiting a high coverage of the H3K9me3 histone mark. This chromatin pattern is enriched with ERV2 elements, which are associated with recent expansions of retrotransposons. Furthermore, genomic regions encompassing this subcompartment have been found to engage in extensive trans-interactions that are exclusive to neurons and not observed in NeuN-negative cells. The authors further demonstrated that SETDB1 histone methyltransferase deficiency, coupled with a significant decrease in H3K9me3 levels at ERV2 sequences in neuronal chromatin, leads to an increase in ERV2 transcription. This increase in transposon expression was primarily observed in a specific chromatin subcompartment (referred to as B2), while other subcompartments did not exhibit significant changes in transposon expression. Additionally, there was a notable decrease in cis-interactions between B2 and A2 (one of the active subcompartment types). This reduction in interactions between subcompartments was associated with an activation of gene expression within the A2 subcompartment. Importantly, only a few genes up-regulated following *Setdb1* ablation were marked with H3K9me3 in control cells, suggesting that their activation was likely due to the loss of interactions with the B2 subcompartment rather than the direct loss of H3K9me3 at a gene body [22]. Therefore, isolating ERV2-containing chromatin regions into specific subcompartments may act as an additional mechanism to prevent these genetic elements from becoming active in neurons.

In addition to variations in contact strength within compartments, a significant compartmental switching has been observed between neuronal and NeuN-negative cells [20]. Compartment switching is potentially associated with cell-type-specific gene expression because regions that switch from compartment B to A in neuronal cells exhibit higher levels of expression in neurons, whereas regions that switch from compartment B to A in NeuN-negative cells show increased expression in glial cell types such as oligodendrocytes and astrocytes [20]. Compartment switching down-regulates genes essential for myelination and oligodendrocyte differentiation in neurons but not in NeuN-negative cells, which is important for functional divergence between these cell types. Furthermore, the repression of critical neurodevelopmental genes involved in axon guidance and synapse organization emphasizes the importance of compartment switching during neuronal maturation and establishment of distinct neuronal identities [19].

The potential functional relevance of compartmental switching is further supported by a recent study of the mouse brain at the single-cell resolution [23]. In particular, regions undergoing compartmental switching overlap with genes that are crucial for neuronal function and exhibit increased expression during brain development. These observations imply that substantial chromosomal conformation changes may be established in early development and persist to maintain cell-type specificity in the adult brain [23]. Collectively, these findings underscore the presence of cell-type-specific differences in large-scale chromatin organization and highlight the emerging role of compartment switching in the fine-tuned regulation of gene expression, which is crucial for maintaining the distinct cellular identities in the brain.

### TADs in neurons and non-neuronal brain cells

TADs play a critical role in regulating gene expression by facilitating or inhibiting interactions between genes and regulatory elements [29]. While the boundaries of most TADs remain consistent across different cell types [5], the identification of cell-type-specific boundary groups, particularly in brain cells, suggests their involvement in fine-tuning gene regulation and establishing unique expression patterns for each cell type. Increasing evidence indicates that the formation of TAD boundaries is mediated by the arrest of loop extrusion at convergent CTCF binding sites. Indeed, several studies have focused on disrupting key players of the loop extrusion model and have provided compelling evidence for its role in shaping genome features at the finest level of organization [30, 31]. This model is likely a key mechanism for TAD formation in brain cells as well. TAD boundaries in neurons, both specific to neuronal cells and shared with NeuN-negative ones, show an enrichment of chromatin states associated with active histone modifications, particularly H3K4me3 and H3K27ac [19]. These modifications are known to be associated with transcription start sites (TSSs), which are important sites for cohesin recruitment [32]. Furthermore, an analysis of the mouse genome architecture at the single-cell level has revealed a higher likelihood of TAD boundary formation at the TSSs and transcription termination sites (TTSs) of long genes involved in neuronal pathogenicity and essential functions [23]. In line with these observations, it has been shown that TAD boundaries in neurons are enriched with genes that exhibit differential expression compared to NeuN-negative cells and are related to neuronal functions [15]. Increased histone acetylation at TAD boundaries in neurons correlates with large-scale structural features. Specifically, boundaries with high levels of acetylation may interact more frequently, potentially leading to an increase in short-range A–A interactions observed in this cell type. This mechanism seems to parallel observations made in *Drosophila*, where chromatin is characterized by the clustering of TAD boundaries that are enriched with active chromatin marks. In *Drosophila*, such clustering is thought to amplify the local concentration of active transcription machinery at nearby enhancers [33].

However, emerging evidence suggests that TAD boundaries shared by neurons and NeuN-negative cells exhibit an increase in the Polycomb-repressed chromatin state in NeuN-negative cells [15, 19], which is associated with the H3K27me3 histone mark. Previous research in developing oocytes has suggested the significance of the H3K27me3 mark in the formation of cohesin-independent compartmental domains that are thought to be maintained through phase separation [10, 34]. Therefore, it can be suggested that the phase separation of active and inactive chromatin might play a role in the formation of these domains in NeuN-negative cells. Additionally, the presence of inactive marks at shared TAD boundaries in NeuN-negative cells may serve as a mechanism suppressing the expression of neuron-specific genes. Indeed, the distribution of chromatin states at these boundaries suggests that TAD boundaries in brain cells potentially align with neuronal, rather than non-neuronal, transcriptional activity. This alignment may be due to the fact that neurons differentiate earlier in lineage commitment [35], with TAD boundaries already established in precursor cells and conserved throughout brain cell development [19, 36, 37]. Supporting this, neural cells generated from human pluripotent stem cells (hPSCs) typically arise under conditions devoid of morphogens or in the presence of morphogen inhibitors, following a default developmental pathway [38]. However, the absence of direct evidence emphasizes the need for a more detailed analysis of TAD boundaries at different stages of brain development. Such investigations are crucial to support the hypothesis that the TAD boundaries in brain cell precursors are intricately associated with neuronal transcriptional activity, thereby providing a deeper understanding of the regulatory mechanisms influencing brain development.

### Cell-type-specific chromatin loops in brain cells

Chromatin loops, which represent the finest scale of chromosome organization, also display cell-type-specific characteristics. The hypothesis of a more prominent loop extrusion mechanism in neurons is supported by the increased number of enhancer-promoter loops in neurons compared to NeuN-negative cells. Furthermore, repressor-promoter loops, which also occur in greater abundance in neurons, have been demonstrated to span larger genomic distances than enhancer-promoter loops. Consistent with these findings, there is a general increase in loop length observed in neurons [39]. The study conducted by Calderon et al. has provided further insights into the role of cohesin in cell-specific functioning, demonstrating that the deletion of the cohesin subunit *Rad21* during neuronal differentiation disrupts maturation and significantly down-regulates genes associated with neuronal functions [40]. This research also suggests that the reliance on loop extrusion for enhancer-promoter interactions varies. Specifically, secondary response genes, which are involved in long-range chromatin contacts in neurons, are cohesin-dependent, in contrast to short-range interactions of immediate early genes. This aligns with studies showing that the depletion of *Nipbl* leads to the down-regulation of genes involved in long-distance interactions [25]. Therefore, it can be suggested that a higher level of cohesin-related proteins might enhance neuron-specific long-range chromatin interactions, including those between enhancers and promoters. Supporting this hypothesis, a study conducted by Hsieh et al. has demonstrated that only the longest enhancer-promoter and promoter-promoter non-cohesin loops are weakened upon CTCF/cohesion depletion [41]. While future research is needed to fully understand the underlying mechanisms, these findings highlight the importance of cohesin and cohesin-dependent mechanisms in maturation of neurons and the maintenance of their normal functioning by regulating chromatin loop formation.

Given that enhancer-promoter interactions have been found to regulate cell-type-specific gene expression [42], expression-related variations in chromatin loops have also been observed. In particular, enhancer-promoter interactions specific to neurons or NeuN-negative cells are enriched with genes that are characteristic of these cell types. Furthermore, cell-type-specific enhancer-promoter interactions, as well as differential H3K27ac peaks, are enriched with synaptic and axonal genes in neurons, and actin-based motility genes in NeuN-negative cells [43]. The significance of enhancer–promoter interactions in determining cell fate has been further highlighted by advancements in methodologies that enable more detailed investigation of these interactions. Such studies indicate that dynamic enhancer-promoter loops can serve as reliable biomarkers for neuronal differentiation [44]. Therefore, enhancer–promoter interactions are involved in a dynamic interplay between genome architecture and transcription, reflecting a distinct mechanism of cell-type-specific gene regulation. While the exact mechanisms underlying the formation of differential loops are not yet fully understood, an analysis of various brain cell types suggests that these interactions may be mediated by mechanisms different from those observed in constitutive loops. In particular, motifs of cell-type-specific transcription factors are enriched at the anchor sites of these differential loops, while loops involving housekeeping genes exhibit enrichment of CTCF [21]. Thus, the formation of cell-type-specific enhancer-promoter interactions may involve a more intricate process that is separate from the CTCF-mediated mechanisms.

Another aspect of CTCF involvement in loop formation is demonstrated in a study conducted on mouse hippocampal neurons using a model of a single epileptic seizure episode [45]. Upon induced activation, CTCF loops do not exhibit alterations compared to the naive state. Instead, the CTCF loop-independent interactions become prominent. Specifically, there is an enhancement of interactions between proximal extragenic differentially accessible regions and TSSs of activity-induced genes, as well as between their TSSs and TTSs. Collectively, these observations suggest that while CTCF loops play a role in maintaining cell-type specificity, they may contribute less to the dynamic response induced by environmental changes [45].

### FIREs as distinct features of chromatin organization

In addition to the conventional levels of chromatin organization, recent research has identified additional units of chromatin architecture. One of these units is Frequently Interacting REgions (FIREs), also known as interaction hotspots, which display higher levels of local chromatin interactions. FIREs are functionally and spatially distinct from TADs and chromatin loops, as indicated by their unique positioning and proposed role. FIREs are predominantly located in the A compartment, within TADs and chromatin loops [46]. Functionally, FIREs are thought to play a crucial role in the regulation of cell-type-specific gene expression, as evidenced by their enrichment with cell-type-specific enhancers and differential H3K27ac peaks in both neuronal and NeuN-negative cells [43, 46]. Notably, FIREs specific to NeuN-negative cells were enriched with genes involved in myelination, cell-type-specific differentiation, and oligodendrocyte differentiation, while neuron-specific FIREs overlapped with genes involved in synaptic function. The link between differential FIREs and gene activity was further validated through single-cell RNA sequencing analysis. This analysis revealed that genes associated with neuron-specific FIREs were primarily active in neurons, while genes associated with NeuN-negative FIREs were highly expressed in all types of NeuN-negative cells, especially oligodendrocytes [20].

Further evidence elucidating the role of FIREs in regulating specific gene expression was obtained from experiments conducted on SATB2-deficient mouse neurons. SATB2 encodes a DNA-binding protein that is highly conserved in its primary sequence across different vertebrate species [47]. Due to its important role in transcription regulation and chromatin reshaping [48], it is expected that targeted deactivation of SATB2 would lead to significant changes in chromatin organization. Indeed, the disruption of SATB2 not only affected different chromatin structures but specifically altered the distribution of FIREs. Intriguingly, FIREs that disappeared after SATB2 disruption contained genes associated with neurological functions, including synapses, behavior, learning, memory, and cognitive processes [49]. These observations, combined with the identification of cell-type-specific chromatin characteristics, suggest that differential FIREs are essential for fine-tuning gene expression regulation in order to meet the specific needs of different cell types in the brain.

### Polycomb-mediated interactions in brain cells

In recent studies, PcG proteins have emerged as crucial modulators of the three-dimensional genome organization, forming Polycomb-mediated TADs [10, 11] and mediating long-range DNA interactions [12–15]. However, they were initially recognized for their critical role in maintaining gene repression, a function that has been extensively studied in the context of development [50, 51]. In particular, PcG proteins regulate expression of Hox gene families in mice [52]. Moreover, a significant proportion of genes repressed by PcG proteins are transcription factors that promote cell differentiation, including neuronal factors, underscoring the importance of PcG proteins in neurogenesis [51]. Accordingly, several studies have demonstrated the involvement of PcG proteins in the transition from embryonic stem cells (ESCs) to neural progenitor cells (NPCs). For instance, the Polycomb domains characterized by Kundu et al. are responsible for repressing clusters of genes, including Hox and Pax gene families, during this transition [11]. Moreover, Polycomb activity not only regulates NPCs maturation but also the subsequent differentiation of neurons and glia [53]. Interestingly, during neurogenesis in mice, PRCs undergo multiple reassemblies with varying stoichiometries, which influence their binding dynamics [54]. Indeed, in different assemblies, PcG proteins can repress different factors and act either in a pro-neuronal or pro-glial manner [53].

Evidence suggests that PcG proteins remain active in adult neurons even after neurogenesis [15, 55]. In mature neurons, Polycomb complexes are formed with a slightly different subunit composition than during differentiation. For example, the EZH2 subunit of the PRC2 core is more abundant during the early stages of neurogenesis, whereas EZH1 is observed in mature neurons [55]. This data is supported by transcriptomic experiments. Significant changes in the expression of Polycomb subunits over time have been observed in hPSCs differentiated into TBR1+ neurons over 100 days [56]. The PRC2 subunit EZH2 and PRC1 subunit RNF2 are mostly expressed during the early stages of neurogenesis, while the expression of the PRC1 subunit PCGF5 is increased in mature neurons. Importantly, the inhibition of EZH2 in these cell cultures leads to a premature triggering of the maturation of both neurons and astrocytes. *In vivo*, the disruption of PRC2 has been shown to result in fatal neurodegeneration in mouse models [55]. Collectively, these findings demonstrate the importance of the multisubunit nature of PRC1 and PRC2 complexes for the fine-tuning of their structures, which is essential for orchestrating proper developmental processes.

Beyond their conventional repressive role, PcG proteins are also implicated in mediating long-range DNA interactions, particularly in brain cells. Such interactions have first been observed in NPCs differentiated from mouse ESCs [13, 14]. Of note, their formation appears to be independent of the CTCF or cohesin presence [57]. A recent study conducted by Pletenev et al. has shed light on the role of PcG proteins in adult human neurons by revealing pronounced long-range PcG-mediated chromosomal interactions (Fig. 2C), which are notably absent in NeuN-negative cells [15]. PcG-mediated interactions form high-order loop networks, distinguishing neurons from other cell types. The anchors of these loops are enriched with ChIP-seq peaks representing the H3K27me3 histone modification and PcG subunits. These anchors also overlap with the promoter regions of many transcription factors involved in development and neurogenesis [13, 57]. A comparison of several Hi-C datasets has shown the highest abundance of PcG-mediated interactions in ESCs and NPCs, which is in line with previous transcriptome analyses [56, 57].

Despite these findings, the functional role of PcG proteins in developing and mature neurons remains largely unexplored. It is particularly interesting to consider the biological consequences of such a large difference in the abundance of PcG-mediated loops between neurons and NeuN-negative cells. Hypothetically, by forming multivalent repressive hubs, PcG proteins may down-regulate the expression of developmental transcription factors and other genes with functions specific to NeuN-negative cells, contributing to the narrow specialization of neurons. In addition, preliminary data suggests that neuronal PcG-mediated loops may differ between neuronal subtypes [15]. Maintaining PcG-mediated interactions in mature neurons may therefore provide a separation between excitatory and inhibitory fates.

### The role of cell-type-specific chromatin architecture in brain-related disorders

The three-dimensional organization of chromatin plays a pivotal role in brain development and function [58, 59]. Alterations in this intricate architecture can impact gene expression and potentially contribute to the development of psychiatric disorders. Research conducted by Rajarajan et al. has shown that the differentiation of neural progenitor cells into neurons or astrocyte-like glial cells is accompanied by significant changes in the three-dimensional genome structure. This reconfiguration involves the selective disruption and establishment of chromosomal loops, which are critical for regulating gene activity during cellular maturation [39]. Furthermore, this study revealed that neurons, in contrast to astrocyte-like glial cells, display a higher number of chromosomal interactions at genomic loci associated with schizophrenia (SZ) risk variants. These findings suggest that a neuron-specific chromatin spatial organization pattern may contribute to SZ susceptibility.

Consistent with these observations, a comparative study between neurons and NeuN-negative cells has revealed that differential chromosomal loops in both mature and fetal neurons are significantly associated with traits of SZ and bipolar disorder (BD) [19]. Intriguingly, enhancers transcribed specifically in neurons, compared to those in NeuN-negative cells, were highly enriched with risk variants for the same neuropsychiatric conditions [60]. This finding is supported by similar observations from gene expression profiling and chromatin accessibility analyses conducted in the developing human cerebral cortex [61]. Additionally, this study has uncovered previously unknown associations between NeuN-negative cells and neuropsychiatric conditions, such as the link between oligodendrocytes and Tourette syndrome, as well as the association between astrocytes and obsessive-compulsive disorder. Notably, an enrichment of non-psychiatric, immune-related features was exclusively observed in microglia. Different types of neurons possess unique nuclear chromatin patterns and epigenetic landscapes, which systematically vary across different cortical areas. Disruptions in these epigenetic states have been linked to the pathophysiology of neurodevelopmental disorders, such as autism spectrum disorders [62].

However, insights into the relationship between the epigenetic landscape of neurons and psychiatric disorders have been derived from studies conducted on healthy controls, relying on heritability analysis and assessment of the enrichment of disease-associated SNPs. A new wave of studies is currently underway, focusing on direct comparisons between affected and control samples. For example, an analysis of the H3K27ac profile in neuronal cells recently discovered an increased number of SNPs associated with SZ and BD within hyperacetylated but not hypoacetylated peaks specifically in diagnosed samples. In addition, this analysis introduced a novel concept of acetylated regions, termed cis-regulatory elements (CRDs), which are closely related to the spatial structure of chromatin. Thus, the proximity and connectivity of TADs, as measured by pairwise Euclidean distances between TADs from hyperacetylated CRDs located within the A-compartment and associated with SZ, were significantly higher compared to the 3D connectivity of all CRDs [63]. Another similar study investigating changes in chromatin accessibility confirmed the previously established distinction between neurons and NeuN-negative cells in the light of their contribution to psychiatric disorders. Indeed, in contrast to neurons, NeuN-negative cells exhibited significantly fewer changes in chromatin accessibility profiles when comparing individuals with psychiatric disorders and healthy controls [64].

Taken together, these findings emphasize the significance of cell-type-specific chromatin configurations in investigating the genetic mechanisms that contribute to neurological disorders. However, the available data on these configurations in neurological disorders remains limited and mainly focuses on enhancer-promoter interactions and chromatin accessibility.

## Conclusion

Despite the central role of three-dimensional genome organization in regulating brain function, research in this area is limited by the complexity of brain anatomy and the heterogeneity of its cells. However, the emergence of high-throughput sequencing and improved techniques for studying the spatial organization of the genome enables comprehensive analyses of brain samples. Recent research has begun to uncover previously unknown aspects of chromatin architecture in different types of brain cells and the role of chromatin organization in the regulation of gene expression. Despite these insights, a comprehensive understanding of chromatin organization across major brain cell types remains elusive. The key findings of recent research on conventional levels of chromatin organization suggest a potential increase in cohesin or cohesin-related proteins in neuronal cells, warranting quantitative proteomic studies to validate this hypothesis. Moreover, emerging research on additional layers of chromatin organization, such as PcG-mediated interactions, calls for detailed microscopy studies to elucidate the presence and spatial distribution of neuron-specific Polycomb networks. Additionally, conducting comparative analyses between different brain regions could be valuable for uncovering the relationship between genome architecture and functional diversity in these regions. Considering the existing research on the differences in chromatin organization between neurons and NeuN-negative cells, there is a strong need for future studies that focus on individual cell types, aiming to generate Hi-C data for specific subtypes of neurons and NeuN-negative cells, known for their unique characteristics and functions. These analyses are particularly relevant when comparing individuals with and without brain-related conditions, as understanding the exact causes of the observed differences could pave the way to novel therapeutic strategies. Ultimately, delving into the mechanisms behind the distinct chromatin architectures in brain cells is essential for unraveling the complex epigenetic landscape that shapes both normal brain function and abnormal conditions.

## Data Availability

No datasets were generated or analysed during the current study.
